# The CCR2 Inhibitor Propagermanium Attenuates Diet-Induced Insulin Resistance, Adipose Tissue Inflammation and Non-Alcoholic Steatohepatitis

**DOI:** 10.1371/journal.pone.0169740

**Published:** 2017-01-11

**Authors:** Petra Mulder, Anita M. van den Hoek, Robert Kleemann

**Affiliations:** 1 Department of Metabolic Health Research, Netherlands Organization for Applied Scientific Research (TNO), Zernikedreef 9, Leiden, The Netherlands; 2 Department of Vascular Surgery, Leiden University Medical Center, Leiden, The Netherlands; University College London, UNITED KINGDOM

## Abstract

**Background and aim:**

Obese patients with chronic inflammation in white adipose tissue (WAT) have an increased risk of developing non-alcoholic steatohepatitis (NASH). The C-C chemokine receptor-2 (CCR2) has a crucial role in the recruitment of immune cells to WAT and liver, thereby promoting the inflammatory component of the disease. Herein, we examined whether intervention with propagermanium, an inhibitor of CCR2, would attenuate tissue inflammation and NASH development.

**Methods:**

Male C57BL/6J mice received a high-fat diet (HFD) for 0, 6, 12 and 24 weeks to characterize the development of early disease symptoms of NASH, i.e. insulin resistance and WAT inflammation (by hyperinsulinemic-euglycemic clamp and histology, respectively) and to define the optimal time point for intervention. In a separate study, mice were pretreated with HFD followed by propagermanium treatment (0.05% w/w) after 6 weeks (early intervention) or 12 weeks (late intervention). NASH was analyzed after 24 weeks of diet feeding.

**Results:**

Insulin resistance in WAT developed after 6 weeks of HFD, which was paralleled by modest WAT inflammation. Insulin resistance and inflammation in WAT intensified after 12 weeks of HFD, and preceded NASH development. The subsequent CCR2 intervention experiment showed that early, but not late, propagermanium treatment attenuated insulin resistance. Only the early treatment significantly decreased Mcp-1 and CD11c gene expression in WAT, indicating reduced WAT inflammation. Histopathological analysis of liver demonstrated that propagermanium treatment decreased macrovesicular steatosis and tended to reduce lobular inflammation, with more pronounced effects in the early intervention group. Propagermanium improved the ratio between pro-inflammatory (M1) and anti-inflammatory (M2) macrophages, quantified by CD11c and Arginase-1 gene expression in both intervention groups.

**Conclusions:**

Overall, early propagermanium administration was more effective to improve insulin resistance, WAT inflammation and NASH compared to late intervention. These data suggest that therapeutic interventions for NASH directed at the MCP-1/CCR2 pathway should be initiated early.

## Introduction

Non-alcoholic fatty liver disease (NAFLD) is the most common cause of chronic liver disease worldwide [1]. NAFLD encompasses a spectrum of liver conditions ranging from steatosis (NAFL) to steatosis with hepatic inflammation (non-alcoholic steatohepatitis, NASH) which can lead to liver fibrosis, cirrhosis and liver-related mortality [2]. The rise in prevalence of NAFLD parallels the dramatic increase in obesity [1]. It has been postulated that the chronic, low-grade inflammatory state that characterizes obesity may play a central role in driving the development of NASH [3]. Thus, anti-inflammatory treatments may have therapeutic potential to reduce obesity-associated NASH development.

The expanding white adipose tissue (WAT) in obesity may constitute an important source of inflammation during the development of NASH [4]. Many studies have demonstrated that WAT inflammation in obese subjects is promoted by infiltrating macrophages [5, 6]. Recently, we have shown that surgical excision of inflamed WAT can attenuate NASH, providing first evidence for a causal role of WAT in NASH development [7].

The chemokine monocyte chemoattractant protein (MCP)-1 and its receptor C-C chemokine receptor-2 (CCR2) play a pivotal role in the recruitment of macrophages/monocytes to the sites of inflammation both in WAT [8, 9] as well as in liver [9–11]. For instance, mouse models with genetic deletion of MCP-1 or CCR2 have shown that these factors control the infiltration of macrophages into WAT and are crucial for the development of insulin resistance and hepatic steatosis in high-fat diet (HFD)-induced obese mice [12, 13]. It also has been reported that CCR2-deficient mice have decreased accumulation of inflammatory cells in liver [10, 14]. Furthermore, previous studies have shown that the CCR2 inhibitor propagermanium can prevent insulin resistance and steatosis in db/db mice [15] and wild-type mice [16]. However, *prophylactic* administration was used in the latter experiments and it therefore remains unknown whether therapeutic intervention with propagermanium in the ongoing disease process of NASH, i.e. reflecting the clinical setting, will be effective. To answer this question, we first examined the development of disease symptoms insulin resistance, WAT and liver inflammation in time, in order to define adequate time points for propagermanium intervention. To do so, male C57BL/6J mice were fed a high-fat diet (HFD) for 0, 6, 12 and 24 weeks and insulin resistance was characterized by hyperinsulinemic-euglycemic clamp, and WAT and liver inflammation by histology. In a subsequent *intervention* study, we investigated whether propagermanium treatment, started at different time points in the disease development (early vs. late), would attenuate NASH development in mice with manifest disease symptoms.

## Materials and Methods

All animal experiments were approved by the institutional Animal Care and Use Committee of the Netherlands Organization of Applied Scientific Research (Zeist, The Netherlands; approval number DEC3412) and were in compliance with European Community specifications for the use of laboratory animals. Male 9-week old wild type C57BL/6J mice were obtained from Charles River Laboratories (L'Arbresle Cedex, France) and were kept on chow control diet during a 3-week acclimatization period until the start of the experiment (R/M-H, Ssniff Spezialdieten GmbH, Soest, Germany). Mice were housed in a temperature-controlled room with a regular 12-h light/dark cycle, with ad libitum access to food and water.

### Time-course hyperinsulinemic–euglycemic clamp experiment

A group of mice (n = 12) was used as reference to define the condition prior to HFD feeding (at t = 0). Three groups (n = 12 each) were treated with a high-fat diet (HFD) for 6, 12 and 24 weeks (D12451, Research Diets Inc., New Brunswick, USA) to determine the development of whole-body and tissue-specific insulin resistance. A hyperinsulinemic-euglycemic clamp analysis was performed in all groups as described previously [17]. Briefly, after an overnight fast, mice were anesthetized with 6.25 mg/kg vetranquil (Sanofi Santé Nutrition Animale, Libourne Cedex, France), 6.25 mg/kg dormicum (Roche, Mijdrecht, The Netherlands) and 0.3125 mg/kg fentanyl (Janssen-Cilag, Tilburg, the Netherlands) and an infusion needle was placed in one of the tail veins. Basal rates of glucose turnover were determined by administering a primed (0.72 μCi/μl), continuous (1.2 μCi/h) infusion of [^14^C]-glucose for 60 minutes. Subsequently, the hyperinsulinemic condition was started with a primed (4.1 mU) continuous (6.8 mU/h) infusion of insulin (Actrapid, Novo Nordisk, Alphen a/d Rijn, The Netherlands). A variable infusion of 12.5% D-glucose was used to maintain euglycemia (measured at 10 min intervals via tail bleeding using the ‘Freestyle glucose measurement system’ from Abbott (Abbott Park, IL, USA). Blood samples (75 μl) were collected during the basal period (after 50 and 60 minutes) and during the clamp (hyperinsulinemic) period (after 70, 80 and 90 minutes) for determination of plasma glucose, insulin and ^14^C-glucose specific activities. To assess insulin-mediated glucose uptake in white adipose tissue (WAT), 2-deoxy-D-[3H] glucose (2-DG glucose; Amersham, Little Chalfont, UK) was administered as a bolus (1 μCi), 40 minutes before the end of the clamp experiment. After the clamp, mice were sacrificed, plasma was collected by heart puncture and liver and adipose tissue were isolated. One part of the tissues was fixed in formalin and paraffin-embedded for histological analysis, another part was frozen in liquid nitrogen for subsequent analysis.

### CCR2 inhibitor intervention experiment

Mice were matched based on body weight and fasting insulin plasma concentration and divided into a chow control diet (n = 5) control group and a HFD treatment group (n = 45). Mice on HFD were matched again after 6 weeks of HFD feeding and divided into three groups (n = 15 each). One group continued on HFD until the end of the study (24 weeks). The early intervention group received HFD supplemented with propagermanium from 6 weeks onwards, for a total of 18 weeks (HFD+Pro_6w, 0.05% w/w; Sigma Aldrich, Zwijndrecht, The Netherlands). The late intervention group received HFD supplemented with propagermanium from 12 weeks onwards for another 12 weeks (HFD+Pro_12w). Propagermanium is an organic compound, which has been clinically used for the treatment of hepatitis B virus-induced chronic hepatitis [18]. The current concentration of propagermanium has been previously used [16] and did not show adverse effects. In week 24, all animals were sacrificed by CO_2_ asphyxiation. Serum was collected by heart puncture and major WAT depots and livers were isolated. One part of the tissues was fixed in formalin and paraffin-embedded for histological analysis. Another part was snap-frozen in liquid nitrogen and stored at -80°C for real-time polymerase chain reaction (RT-PCR). Two mice that were resistant to develop diet-induced obesity on HFD (i.e. body weight gain 50% less than group mean) were excluded from the study at sacrifice.

### Body composition

Total body fat was determined using a NMR Echo MRI whole body composition analyzer (EchoMRI LLC, Houston, TX, USA) at the end of the study (week 24).

### Blood and plasma analyses

Blood samples were taken at regular intervals by tail incision after a 5-hour fast. Blood glucose was measured immediately using the ‘Freestyle glucose measurement system’ from Abbott (Abbott Park, IL, USA). Plasma insulin levels (Ultrasensitive mouse insulin ELISA, Mercodia, Uppsala, Sweden) and plasma adiponectin levels (R&D Systems Ltd, Abington, UK) were determined by ELISA. Homeostasis model assessment (HOMA) was used to calculate relative insulin resistance (IR). Five hours fasting plasma insulin and fasting blood glucose values were used to calculate IR, as follows: IR = [insulin (ng/ml) × glucose (mM)]/22.5.

### Histological and biochemical analysis of adipose tissue and liver

Paraffin-embedded cross-sections (5 μm) of adipose tissue were stained with hematoxylin-phloxine-saffron (HPS). WAT inflammation was quantified blindly by counting the number of crown-like structures (CLS) in 3 non-overlapping fields (at x100 magnification) and expressed as number of CLS per mm^2^.

Hematoxylin and eosin-stained (HE) cross-sections (3μm) of the medial liver lobe (lobus medialis hepatis) were scored blindly using an adapted grading method for human NASH [19]. Briefly, two cross-sections per mouse were examined and the level of macrovesicular and microvesicular steatosis was assessed relative to the liver area analyzed and expressed as a percentage. Hepatic inflammation was evaluated by counting the number of inflammatory cell aggregates per field at a x100 magnification (view size of 3.1 mm^2^) in five non-overlapping fields per specimen, and expressed as average number of cell aggregates per field. Biochemical analysis of intrahepatic triglyceride content was determined by high-performance thin-layer chromatography (HPTLC) as previously described [7].

### Gene expression analyses

Liver RNA was extracted using RNA Bee Total RNA Isolation Kit (Bio-Connect, Huissen, the Netherlands) and Ambion Total RNA isolation was used for RNA extraction of WAT (AM1912, Life Technologies, Bleiswijk, The Netherlands). RNA concentration was assessed spectrophotometrically using Nanodrop 1000 (Isogen Life Science, De Meern, the Netherlands) and RNA quality was determined by Lab-on-Chip analysis using 2100 Bioanalyzer (Agilent Technologies, Amstelveen, the Netherlands). cDNA was synthesized from total RNA using a High Capacity RNA-to-cDNA™ Kit (Life Technologies). Gene expression analyses were performed by RT-PCR on an Applied Biosystems 7500 Fast Real-time PCR system. TaqMan® Gene Expression Assays (Life Technologies) were used to detect the expression of the following genes: CD68 (Mm03047340_m1), CD11c (*Itgax*, Mm00498698_m1), Arginase-1 (*Arg1*, Mm00475988_m1), Mcp-1 (*Ccl2*, Mm00441242_m1), Tnf-α (*Tnf*, Mm00443258_m1). Glyceraldehyde 3-phosphate dehydrogenase (*Gapdh*; 4308313), hypoxanthine-guanine phosphoribosyltransferase (*Hprt*; Mm00446968_m1) and peptidylprolyl isomerase F (*Ppif*; Mm00506384_m1) were used as housekeeping genes. Changes in gene expression were calculated using the comparative Ct (ΔΔCt) method and expressed as fold-change relative to HFD control group.

### Statistical analysis

Results are shown as mean±SEM. Significance of difference between groups were statistically analyzed by One-way ANOVA and Tukey post-hoc tests (for normally distributed variables). Non-normally distributed variables were tested by non-parametric Kruskal-Wallis followed by one-sided Mann-Whitney U test using Graphpad Prism software (version 6, Graphpad Software Inc. La Jolla, USA). Correlations were determined by Spearman's rank correlation. Differences were considered significant at *p*<0.05.

## Results

### Insulin resistance in WAT precedes hepatic insulin resistance in diet-induced NASH

**Fig 1:**
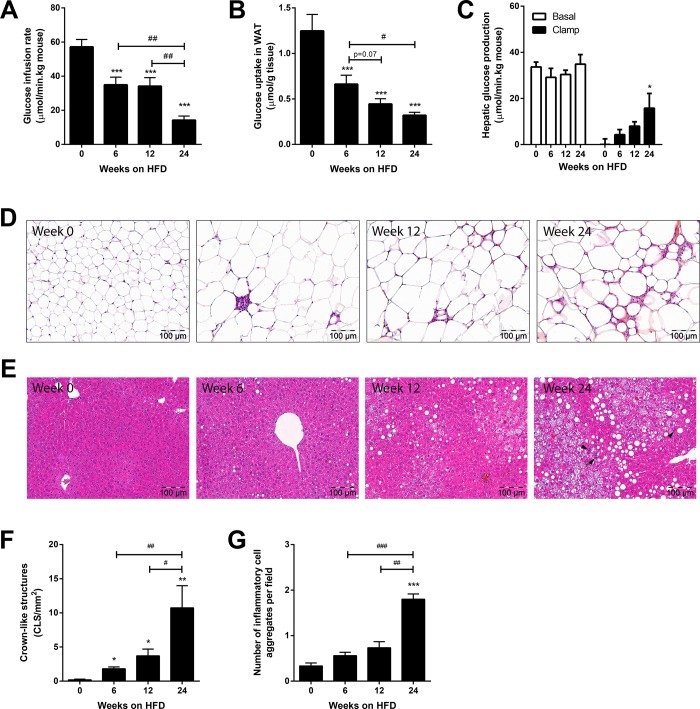
Time course development of insulin resistance, adipose and liver inflammation. Mice were maintained on a high-fat diet (HFD) 0, 6, 12 and 24 weeks prior to clamp experiment to induce insulin resistance. (A) shows HFD-induced decrease in glucose infusion rate (GIR) under hyperinsulinemic conditions, indicating development of whole body insulin resistance. (B) HFD feeding resulted in a gradual decrease of tissue-specific uptake of glucose into white adipose tissue (WAT). (C) HFD feeding increased hepatic glucose production after 24 weeks of HFD, indicating development of hepatic insulin resistance. ‘Basal’ indicates prior to hyperinsulinemic clamp conditions and ‘clamp’ indicates hyperinsulinemic clamp conditions. (D) Representative microphotographs of HPS-stained WAT cross-sections, showing WAT inflammation (i.e. crown-like structures) already after 6 weeks of HFD feeding. (E) Representative microphotographs of HE-stained liver cross-sections, showing NASH development after 24 weeks of HFD feeding (arrow heads indicate clusters of inflammatory cells). (F) Quantitative analysis of crown-likes structures (CLS), demonstrating gradual development of WAT inflammation by HFD feeding. (G) Hepatic inflammation (number of inflammatory cell aggregates per 100x field) was induced after 24 weeks of HFD feeding. All data are mean±SEM, n = 7-11/group. * *p<*0.05 ** *p<*0.01 *** *p<*0.001 compared with t = 0. # *p<*0.05, ###*p<*0.01 compared to week 24.

The development of whole body and tissue-specific insulin resistance was determined by hyperinsulinemic-euglycemic clamps prior to (t = 0) and after 6, 12, 24 weeks of HFD feeding. The glucose infusion rate (GIR), a measure of whole body insulin sensitivity, was markedly lowered after 6 weeks and decreased further at 24 weeks (Fig 1A). The use of radioactive glucose tracers allowed us to determine development of insulin resistance in WAT and liver. The 2-deoxyglucose (2DG) uptake by WAT was already significantly lowered after 6 weeks of HFD feeding, clearly indicating that this tissue had become insulin resistant (Fig 1B), whereas the liver was still insulin sensitive at that time point (Fig 1C). Hepatic glucose production was suppressed by insulin until week 12 of HFD feeding, but thereafter insulins’ inhibitory effect became weaker and hepatic insulin resistance developed after 24 weeks of HFD feeding (Fig 1C). The development of insulin resistance in WAT after 6 weeks of HFD was paralleled by the occurrence of crown-like structures (CLS), a defining characteristic of macrophage-driven WAT inflammation (Fig 1D and Fig 1F). Also, the development of hepatic insulin resistance after 24 weeks of HFD coincided with formation of inflammatory cell aggregates indicating liver inflammation (Fig 1E and Fig 1G). In all, these data demonstrate that insulin resistance in WAT develops rapidly (detectable after 6 and 12 weeks) and precedes liver insulin resistance (24 weeks) and diet-induced NASH.

### Intervention with propagermanium improves insulin resistance independent of obesity

In a separate study, propagermanium interventions were started after 6 weeks (HFD+Pro_6w; early intervention) and 12 weeks (HFD+Pro_12w group; late intervention) of HFD feeding.

**Fig 2:**
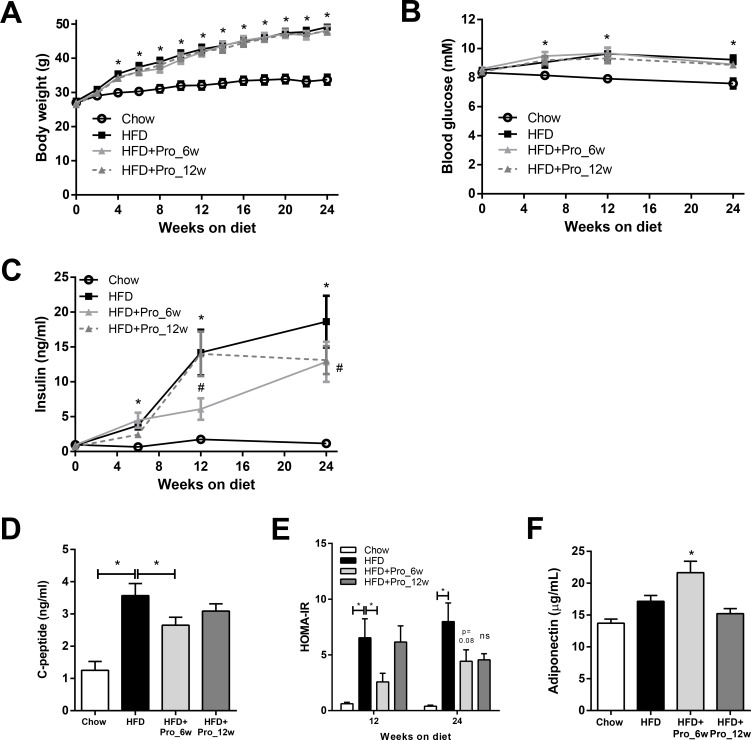
Effects of propagermanium intervention on body weight and metabolic parameters. Mice were fed a high-fat diet (HFD) for 24 weeks or treated with the CCR2 inhibitor propagermanium started after 6 weeks (HFD+Pro_6w, early intervention) or 12 weeks of HFD (HFD+Pro_12w, late intervention). Chow-fed mice were included as a reference. (A) HFD feeding increased body weight compared with chow and was not affected by propagermanium intervention. (B) Fasting blood glucose levels over time. (C) HFD-induced increased fasting plasma insulin levels, which were reduced in HFD+Pro_6w. (D) C-peptide levels were increased at week 24 in HFD-fed mice and attenuated by early propagermanium intervention. (E) HFD-induced increases in HOMA-IR relative to chow were significantly reduced by early, but not late, propagermanium intervention. (F) Plasma adiponectin levels were increased by early propagermanium treatment in week 24. All data are mean±SEM, n = 5 (chow), and n = 13–15 for HFD and intervention groups. * *p<*0.05 (compared with chow) # *p<*0.05 compared with HFD.

The body weight of mice on a HFD increased strongly over time relative to chow control mice (HFD: 49.1±1.0g vs. Chow: 33.7±1.5g, *p*<0.0001; Fig 2A). The increase in body weight in the HFD group was reflected by increased total body fat mass (HFD: 18.6±0.8g vs. Chow: 4.3±1.2g; *p*<0.001). Early and late treatment with propagermanium did not affect body weight (HFD+Pro_6w: 48.2±1.3g and HFD+Pro_12w: 47.7±0.7g, ns; Fig 2A) and total fat mass (HFD+Pro_6w: 18.4±0.8g and HFD+Pro_12w: 17.9±0.4g; ns).

HFD feeding increased fasting blood glucose (9.2±0.3 mM vs. 7.6±0.3 mM in chow; *p*<0.01; Fig 2B), and comparable levels were observed in the intervention groups (HFD+Pro_6w: 8.9±0.2 mM and HFD+Pro_12w: 8.9±0.2 mM, ns; Fig 2B). Moreover, HFD feeding strongly increased fasting insulin levels compared to chow (HFD: 18.6 ±3.7 ng/ml vs. Chow: 1.2±0.3 ng/ml; *p*<0.001). This increase was significantly attenuated in the early intervention group, but not in the late intervention group (Fig 2C). Consistent with this, C-peptide was significantly reduced by the early propagermanium intervention, but not by the late intervention (Fig 2D). Moreover, early intervention with propagermanium significantly lowered fasting HOMA-IR which remained at a lower level until the end of the study (Fig 2E). The anti-inflammatory adipokine adiponectin was increased in plasma at week 24 in those mice receiving early propagermanium treatment (Fig 2F).

Taken together, early intervention, but not late intervention, with propagermanium attenuates insulin resistance and increases plasma adiponectin levels. These effects were independent of body mass and adiposity, suggesting an effect on the inflammatory state of WAT.

### Intervention with propagermanium attenuates WAT inflammation

**Fig 3:**
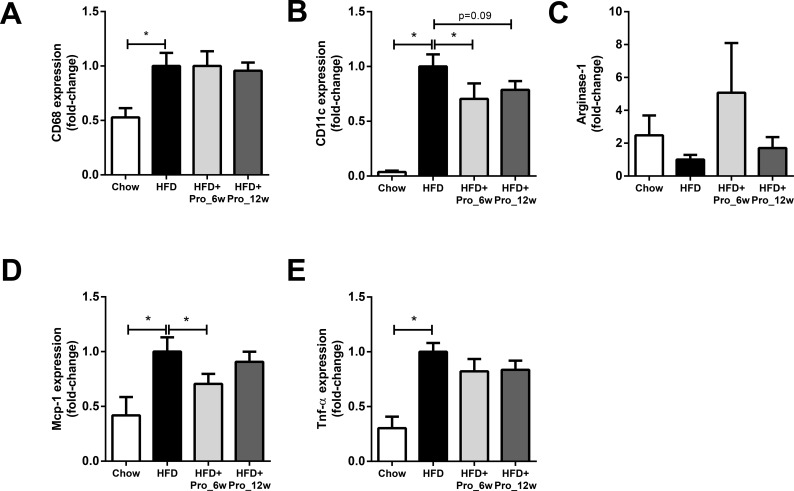
Effects of propagermanium intervention on WAT inflammation markers. (A) HFD feeding increased CD68 expression (general macrophage marker), which was not affected by propagermanium. (B) HFD increased CD11c expression, suggesting increased number of ‘pro-inflammatory’ (M1) macrophages in WAT. (C) Arginase-1 expression (anti-inflammatory M2 macrophage marker) did not differ between the groups. Early intervention significantly lowered HFD-induced expression of (D) Mcp-1 and showed slightly lowered (E) Tnf-α expression levels in WAT. All data are mean±SEM.* *p<*0.05.

MCP-1 and its receptor CCR2 are important for macrophage recruitment [8, 9] as well as polarization of macrophages from an anti-inflammatory M2 toward a pro-inflammatory M1 phenotype in adipose tissue [20]. Both processes contribute to WAT inflammation and have been associated with the development of insulin resistance. As shown in Fig 3A, HFD feeding resulted in increased gene expression of CD68 (a generic macrophage marker) compared to chow (p<0.05), indicating that more macrophages are present in WAT of the HFD-fed mice. Propagermanium interventions did not affect CD68 expression in WAT. A more refined analysis of the macrophage phenotype revealed that HFD treatment strongly increased the gene expression of CD11c, a M1 marker. This increase was significantly reduced by early propagermanium treatment (*p<*0.05 vs. HFD) and tended to be lower in the late intervention group *(p =* 0.09 vs. HFD, Fig 3B). By contrast, expression of arginase-1 (a M2 marker) did not differ between the groups (Fig 3C). In agreement with the observed decrease in expression of CD11c, Mcp-1 expression was significantly attenuated in the HFD+Pro_6w group (*p<*0.05 vs. HFD, Fig 3D). The HFD-induced expression of Tnf-α was diminished in both intervention groups, but this effect did not reach statistical significance (*p =* 0.10; HFD vs. HFD+Pro_6w, Fig 3E).

Collectively, these results indicate that early intervention with propagermanium reduces WAT inflammation as shown by decreased expression of pro-inflammatory M1 macrophage markers.

### Propagermanium intervention attenuates NASH development

**Fig 4:**
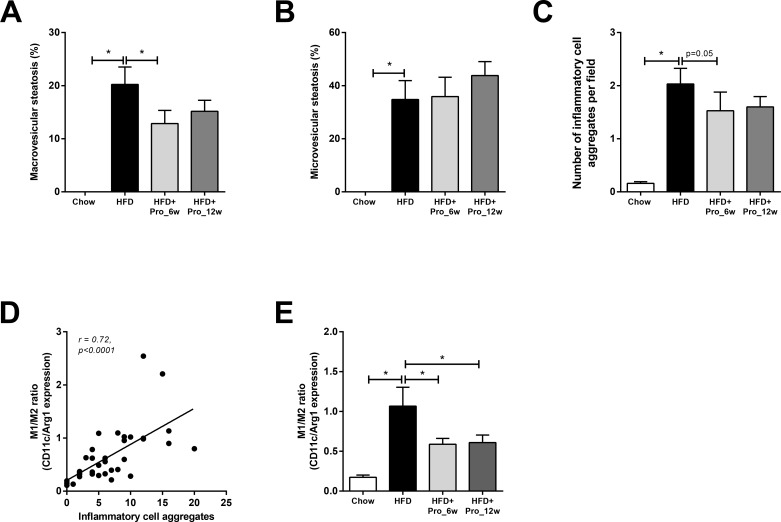
Effects of propagermanium intervention on NASH development. (A) HFD feeding induced pronounced macrovesicular steatosis, which was reduced in HFD+Pro_6w group. (B) Microvesicular steatosis was induced by HFD and was not affected by propagermanium treatment. (C) Quantification of the number of inflammatory cell aggregates per field in the liver (lobular inflammation), which was increased by HFD and tended to be reduced by early propagermanium treatment. (D) Lobular inflammation strongly correlates with the ratio of M1/M2 markers (CD11c/Arginase-1 gene expression). (E) M1/M2 ratio is decreased by propagermanium treatment, indicating a shift towards M2 phenotype. All data are mean±SEM. * *p<*0.05.

Histological analysis revealed a significant decrease in macrovesicular steatosis vs. HFD control group by early intervention only (with -37%, *p<*0.05 for HFD+Pro_6wk and -31%, *p =* 0.14 for HFD+Pro_12 wk; Fig 4A), while microvesicular steatosis was not affected (Fig 4B). Biochemical analysis showed that hepatic triglycerides were slightly reduced by early propagermanium treatment, but did not reach statistical significance (HFD: 168.5±19.0 μg/mg liver protein vs. HFD+Pro_6w: 149.7±22.9 μg/mg liver protein, *p =* 0.27; not shown).

Quantification of inflammatory cell aggregates (a hallmark of NASH) revealed that lobular inflammation was reduced by intervention with propagermanium (Fig 4C), with borderline significant effect of early propagermanium treatment (*p =* 0.05 for HFD+Pro_6wk and *p =* 0.18 for HFD+Pro_12wk, respectively). Lobular inflammation in the HFD group was accompanied by increased hepatic Tnf-α gene expression and increased CD68 gene expression (both, *p<*0.05 vs. chow), which were not affected by propagermanium (not shown). Notably, lobular inflammation positively correlated with the ratio of M1/M2 macrophages (CD11c/Arginase-1 gene expression; r = 0.72, *p<*0.0001, Fig 4D). HFD feeding strongly increased the M1/M2 ratio compared to chow and propagermanium intervention attenuated this HFD effect (Fig 4E). The improvement in M1/M2 ratio by propagermanium is the result of lowered CD11c expression as well as an increase in Arginase-1 expression (S1 Fig). Taken together, propagermanium treatment reduced NASH development with a more pronounced hepatoprotective effect of early intervention.

## Discussion

Accumulating evidence points to an important role of the MCP-1/CCR2 pathway in the development of obesity-associated inflammation in WAT [6, 8, 9, 12, 13] and subsequent development of NASH [9, 10, 14]. Herein, we examined whether therapeutic intervention with the CCR2 inhibitor propagermanium in ongoing disease would attenuate NASH development. We demonstrate that early risk factors of the disease, i.e. insulin resistance and WAT inflammation, can be reduced by propagermanium and that this effect is independent of obesity. Moreover, intervention with propagermanium reduced macrovesicular steatosis and reduced lobular inflammation, indicating an attenuation of NASH development. The effects of propagermanium were more pronounced when the intervention was started early (after 6 weeks of HFD feeding).

NASH is strongly associated with insulin resistance [21, 22]. More specifically, it has been shown the degree of adipose tissue insulin resistance is associated with progressive NASH in patients [23], supporting the view that insulin resistance in WAT is an early disease symptom that may contribute to NASH development [24]. In line with this, our data show that WAT insulin resistance precedes NASH development. Interestingly, hepatic insulin resistance develops much later in time (after 24 weeks of HFD). We observed that development of insulin resistance was paralleled by an increase in inflammatory cells in both, WAT and liver. In addition to changes in number of inflammatory cells, macrophage phenotype may also play a role in the development of insulin resistance.

Macrophages are phenotypically heterogeneous and have been characterized based on their polarization state as ‘pro-inflammatory M1’ or ‘anti-inflammatory M2’ macrophages [25]. The ratio of M1/M2 macrophages may be of particular importance in the development of insulin resistance. For instance, mice lacking M2 polarized macrophages show increased WAT inflammation (e.g. CLS) and worsened insulin resistance [26, 27]. In the present study, we observed a significantly increase in M1 marker CD11c expression in WAT upon HFD feeding and propagermanium attenuated this effect and lowered plasma insulin levels. Notably, therapeutic propagermanium administration did not result in decreased expression of total macrophage marker (CD68) or increased M2 macrophage marker expression, indicating that decreasing pro-inflammatory M1 macrophage content is sufficient to improve obesity-associated insulin resistance. In support of this notion, CD11c depletion in obese mice resulted in a rapid normalization of glucose and insulin tolerance and decreased inflammatory markers in WAT, both at the level of gene transcription and protein expression [28].

The dysregulation of the M1/M2 phenotypic balance in the liver is also emerging as a central mechanism in NASH development [25]. Herein, we observed that HFD feeding increased the ratio M1/M2 macrophage expression in the liver, whereas propagermanium intervention counteracted this effect. Moreover, lobular inflammation positively correlated with the ratio of M1/M2 expression in liver, suggesting that a shift towards M1 macrophages may be critical for NAFLD progression. Consistent with this, Maina and colleagues have shown that hepatic expression of M1 markers (i.e. iNOS, IL-12p40) positively correlated with number of inflammatory foci in livers of C57BL/6 mice [29]. These and our data suggest that suppressing M1 macrophage polarization and/or favoring the differentiation of M2 macrophages in the liver can attenuate NASH development.

Recently, in a randomized controlled clinical trial the effect of the CCR2 antagonist, JNJ-41443532, was examined in obese patients with type 2 diabetes [30]. The administration of this CCR2 antagonist resulted in decreased levels of fasting blood glucose over a 4-week treatment period. HOMA-IR was also lowered in these patients, but did not reach statistical significance. Herein, intervention with propagermanium in obese mice resulted also in reduced plasma insulin and HOMA-IR, but only early treatment showed significant reductions. These data indicate that CCR2 inhibitors may be beneficial to treat insulin resistance, but only when administered early in the disease development, essentially as shown by Tamura and co-workers [15, 16].

Importantly, the effect of CCR2 antagonism on NASH development has not been investigated so far. This study provides experimental evidence that early CCR2 intervention attenuates adipose tissue inflammation in obesity and subsequent NASH development. Late intervention (12 week onwards) showed similar effects, yet not significant. The late intervention was started at a time point were insulin resistance, WAT inflammation and hepatic steatosis were progressing, but without manifest hepatic inflammation. Since the propagermanium treatment already lost effectiveness when started at this time point, it’s unlikely that propagermanium would be able to reverse the disease process when given at a later time point, i.e. when NAFLD has progressed to NASH. Nonetheless, we cannot exclude the possibility that a longer treatment period with propagermanium may be required to achieve a beneficial effect on NASH.

Propagermanium selectively inhibits MCP-1-induced chemotaxis of CCR2-positive monocytes *in vitro* [31]. Accordingly, propagermanium administration has shown to be effective in suppressing inflammatory conditions that are primarily mediated by inflammatory monocytes and macrophages, such as experimental atherosclerosis [32, 33]. However, other chemokine pathways, i.e. chemoattractant CCL5 (RANTES), are also implicated in mediating immune and inflammatory responses in adipose tissue [34, 35] and liver [34, 36, 37], by attracting immune cells including monocytes and T cells. It is thus likely that disease pathways other than MCP-1/CCR2 become upregulated at later stages of disease process (e.g. RANTES/CCR5) and that interventions merely targeting MCP-1/CCR2 become less efficient in treating NASH.

In all, early propagermanium intervention was more effective than late intervention in attenuating insulin resistance, WAT inflammation, and NASH development. The results of this study suggest that therapeutic interventions for NASH directed at the MCP-1/CCR2 pathway should be initiated in an early stage of the disease development in order to be effective.

## Supporting Information

S1 Fig The effect on propagermanium intervention on hepatic gene expression of M1 and M2 macrophage markers. (A) High-fat diet feeding (HFD) increased CD11c expression in liver, suggesting increased number of ‘pro-inflammatory’ (M1) macrophages. Early propagermanium treatment tended to reduce M1 macrophage expression. (B) HFD reduced expression of Arginase-1 in liver, reflecting lower number of anti-inflammatory (M2) macrophages. Propagermanium treatment increased expression of M2 macrophages. All data are mean±SEM. * *p<*0.05.
(TIF)
