# Supplementary figures and images for: The CCR2 Inhibitor Propagermanium Attenuates Diet-Induced Insulin Resistance, Adipose Tissue Inflammation and Non-Alcoholic Steatohepatitis
Source: PLoS One. 2017 Jan 11;12(1):e0169740. doi: 10.1371/journal.pone.0169740 (PMC5226841; doi:10.1371/journal.pone.0169740)

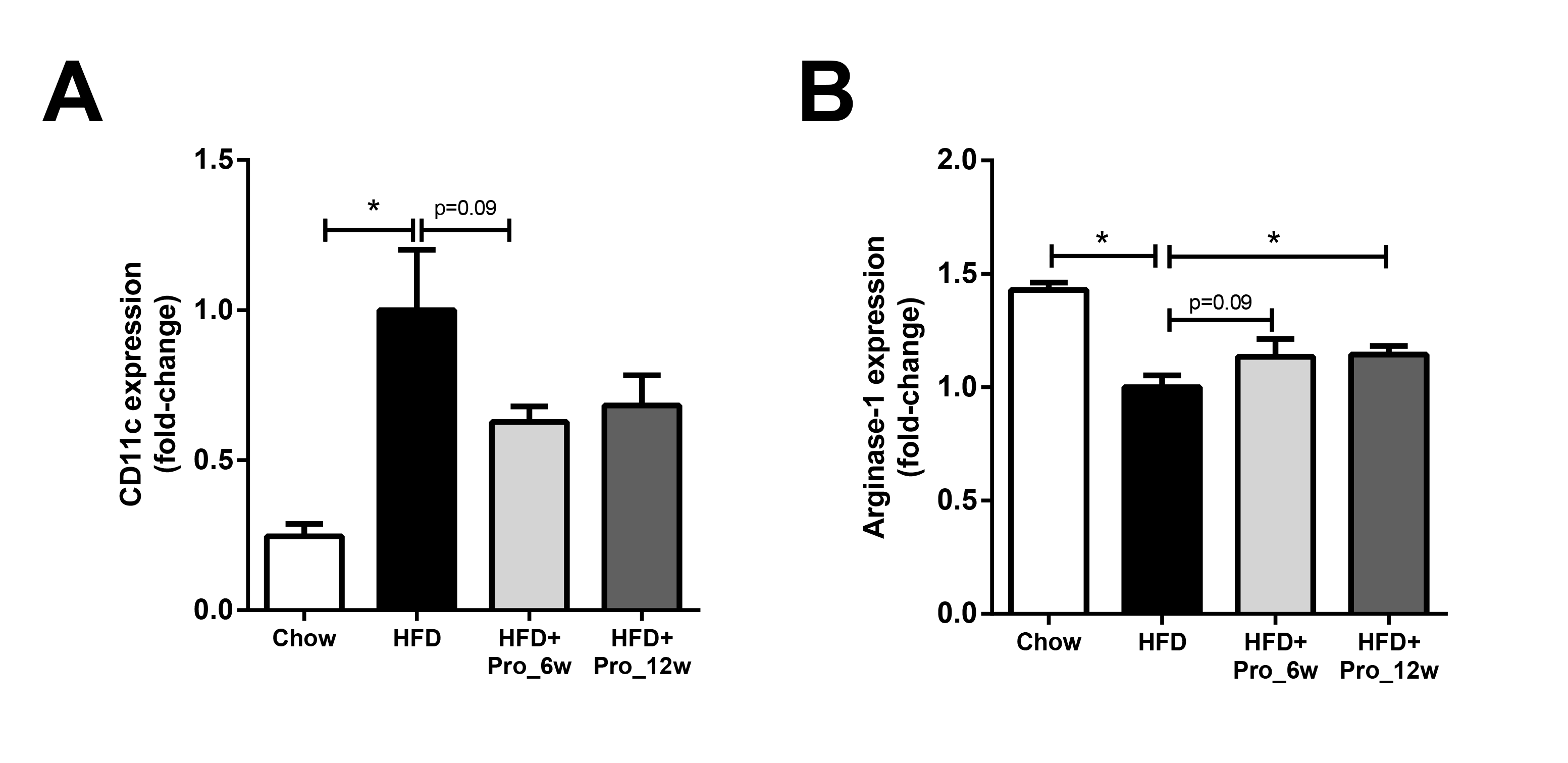

Supplement: S1 Fig — (A) High-fat diet feeding (HFD) increased CD11c expression in liver, suggesting increased number of ‘pro-inflammatory’ (M1) macrophages. Early propagermanium treatment tended to reduce M1 macrophage expression. (B) HFD reduced expression of Arginase-1 in liver, reflecting lower number of anti-inflammatory (M2) macrophages. Propagermanium treatment increased expression of M2 macrophages. All data are mean±SEM. * p<0.05. (TIF) [file pone.0169740.s001.tif]
